# Anti-Inflammatory Effect of Korean Soybean Sauce (Ganjang) on Mice with Induced Colitis

**DOI:** 10.4014/jmb.2404.04020

**Published:** 2024-06-11

**Authors:** Hyeon-Ji Lim, In-Sun Park, Ji Won Seo, Gwangsu Ha, Hee-Jong Yang, Do-Youn Jeong, Seon-Young Kim, Chan-Hun Jung

**Affiliations:** 1 Jeonju AgroBio-Materials Institute, Jeonju-si, Jeollabuk-do 54810, Republic of Korea; 2 Microbial Institute for Fermentation Industry, Sunchang-gun, Jeollabuk-do 56048, Republic of Korea

**Keywords:** Inflammatory bowel disease, traditional fermented Korean soy sauce, ganjang, short-chain fatty acid

## Abstract

Inflammatory bowel disease (IBD), characterized by chronic inflammation of the gut, is caused by several factors. Among these factors, microbial factors are correlated with the gut microbiota, which produces short-chain fatty acids (SCFAs) via anaerobic fermentation. Fermented foods are known to regulate the gut microbiota composition. Ganjang (GJ), a traditional fermented Korean soy sauce consumed worldwide, has been shown to exhibit antioxidant, anticancer, anti-colitis, and antihypertensive activities. However, its effects on the gut microbiota remain unknown. In the present study, we aimed to compare the anti-inflammatory effects of GJ manufactured using different methods and investigate its effect on SCFA production in the gut. To evaluate the anti-inflammatory effects of GJ in the gut, we performed animal experiments using a mouse model of dextran sulfate sodium (DSS)-induced colitis. All GJ samples attenuated DSS-induced colitis symptoms, including reduced colonic length, by suppressing the expression of inflammatory cytokines. In addition, GJ administration modulated SCFA production in the DSS-induced colitis model. Overall, GJ exerted anti-inflammatory effects by reducing DSS-induced symptoms via regulation of inflammation and modulation of SCFA levels in a DSS-induced colitis model. Thus, GJ is a promising fermented food with the potential to prevent IBD.

## Introduction

Inflammatory bowel disease (IBD) is characterized by chronic inflammation of the gut and is caused by multiple factors, such as the immune-mediated system, genetics, and microbial and genetic factors [1-4]. IBD is caused by gut inflammatory responses driven by the activation of the nuclear factor kappa B (NF-κB) and mitogen-activated protein kinase (MAPK) signaling pathways. These inflammatory responses lead to the onset of various symptoms, including rectal bleeding, diarrhea, abdominal pain, and weight loss [5, 6]. Immunosuppressants, 5-aminosalicylic acid, and corticosteroids are commonly used to treat patients with IBD [7]. Recent studies have shown that diet is a major contributor to IBD progression [8]. Dietary fiber, an essential component of the diet, is metabolized by the gut microbiota through anaerobic fermentation, resulting in short-chain fatty acid (SCFA) production [9], which, in turn, is affected by dysbiosis and loss of microbiome diversity. Therefore, an imbalance in SCFA levels causes various diseases, including IBD, colorectal cancer, obesity, hypertension, neurological disorders, pneumonia, and respiratory and metabolic diseases [10]. Increasing evidence suggests that diets such as fermented foods positively modulate the gut microbiota [8, 10]; therefore, fermented foods can be used as therapeutic interventions.

Ganjang (GJ) is a traditional fermented Korean soy sauce used worldwide as a liquid condiment during cooking [11]. GJ is prepared by adding brine to meju (boiled soybean bricks) and fermenting it for at least 3 months to produce a fermented liquid [12]. GJ can be prepared using traditional or commercial methods that differ mainly in the microorganisms used for fermentation [12]. In commercial methods, GJ is produced by fermentation using *Aspergillus oryzae*, whereas in traditional methods, locally available microorganisms are used for fermentation. GJ contains diverse microbial communities and various macromolecules such as amino acids, peptides, saccharides, and organic acids [13]. It exhibits antioxidant, anticancer, anti-colitis, and antihypertensive activities [12]. However, to the best of our knowledge, no study has evaluated the differences in GJ bioactivity owing to differences in the manufacturing methods. Therefore, in this study, we aimed to compare the anti-inflammatory effects of GJ samples produced using different methods through biogenic amine (BA) content and microbial community analysis and investigate the effect of GJ on SCFA production in the gut.

## Materials and Methods

### Preparation of GJ Samples

All GJ samples were provided by the Microbial Institute for Fermentation Industry (Republic of Korea). Traditionally fermented GJ samples produced in Sunchang-gun (GJ1, Republic of Korea), Paju-si (GJ2, Republic of Korea), and Cheongwon-gu (GJ3, Republic of Korea) were used. GJ4 was purchased from a commercial company (Republic of Korea). The GJ samples were diluted to 2% salt concentration using distilled water and then orally administered at 10 ml/kg.

### Determination of Biogenic Amine and Aflatoxin Content

The content of biogenic amines and aflatoxins in the GJ samples was analyzed using high-performance liquid chromatography (Agilent 1200 series) as described previously [14].

### Microbial Community Analysis

The microbial community in the GJ samples was analyzed using next-generation sequencing (NGS) as described previously [15]. Briefly, total DNA from GJ samples was extracted, and the V3-V4 region of the 16S rRNA gene was amplified using targeting primers. Sequencing was performed using the Illumina MiSeq platform (MIFI, Republic of Korea). The obtained sequences were taxonomically classified and analyzed at different levels as described previously [15].

### Animal Studies

Male BALB/c mice (21 ± 1 g, aged 5 weeks, *n* = 32) were purchased from Damool Science (Republic of Korea) and housed under controlled environmental conditions (22°C ± 2°C, 12-:12-h light/dark cycle, and 55% ± 5%relative humidity). All mice were provided a normal diet consisting of a standard laboratory chow (NIH-41 open formula diet; Zeigler Brothers Incorporated, USA). GJ samples were administered orally. After acclimation, the mice were randomly divided into eight groups: (1) water group (water), (2) brine group (water + 2% salt brine), (3) DSS (MP Biomedicals, USA) + brine group (5% DSS + 2% salt brine), (4) DSS + 5-ASA group (5% DSS + 50 mg/kg 5-aminosalicylic acid), (5) DSS + GJ1 group (5% DSS + 2% salinity GJ1), (6) DSS + GJ2 group (5% DSS + 2%salinity GJ2), (7) DSS + GJ3 group (5% DSS + 2% salinity GJ3), and (8) DSS + GJ4 group (5% DSS + 2% salinity GJ4). Fig. 2A presents the animal experimental study plan followed in this study. The symptoms of DSS-induced colitis were evaluated as described previously [15]. After euthanizing the mice, the spleen and intestinal tissues were collected. All animal studies were performed in accordance with the guidelines of the Animal Care Committee of Jeonju AgroBio-Materials Institute (Republic of Korea) and approved by the Animal Care Committee of Jeonju AgroBio-Materials Institute (JAMI IACUC 2022005; Republic of Korea).

### Histological Analysis

For histological analysis, paraffin-embedded colon tissue sections (4-μm-thick) were stained with Alcian blue and hematoxylin and eosin (H&E), and colon tissue damage was scored as described previously [16].

### Measurement of Inflammatory Cytokine Levels

The concentrations of inflammatory cytokines in the serum were determined using a commercial ELISA kit (R&D Systems, USA) according to the manufacturer’s protocol.

### Measurement of Protein Levels

The expression levels of the following proteins in colon tissue samples were determined using specific antibodies: COX-2 (Cell Signaling Technology (CST), USA), iNOS (CST), phospho-p65 (CST), p65 (CST), phospho-p38 (CST), p38 (CST), phospho-ERK (CST), ERK (CST), phospho-JNK (CST), JNK (CST), occludin (CST), ZO-1 (Abcam, UK) and β-actin (CST). The protein concentrations of the homogenates were determined using the BCA protein assay (Bio-Rad Laboratories, USA), and equal amounts of samples were loaded onto SDS-PAGE gels. Images were acquired using Amersham Imager 600 (GE Healthcare, UK).

### Quantitative Real-Time Polymerase Chain Reaction

Total RNA from mouse colon tissues was isolated using an RNA extraction reagent (GeanAll, Republic of Korea) and quantified using a NanoDrop system (Thermo Fisher Scientific, Inc., USA). cDNA was synthesized using a 2× RT reagent kit (Biofact, Republic of Korea). Quantitative real-time polymerase chain reaction (qPCR) was performed using SYBR Green qRT-PCR Master Mix (Biofact) with a real-time PCR system (Bio-Rad Laboratories).

### Immunohistochemical Staining

For immunohistochemical (IHC) staining, paraffin-embedded tissue sections (4-μm-thick) were deparaffinized and rehydrated, and endogenous peroxidase activity was blocked as described previously [16]. Subsequently, the tissue sections were pre-blocked with 4% BSA and incubated overnight at 4°C with anti-ZO-1 (CST) and anti-occludin antibodies (CST). The incubation and substrate reactions were performed using the Anti-Rabbit Envision Plus polymer kit (Dako). IHC results were acquired using a digital slide scanner (Motic, USA).

### SCFA Analysis

Fecal samples were homogenized in hydrochloric acid and isobutanol. The mixture was vigorously vortexed for 10 min and then centrifuged (11,269 g, 5 min) to yield the SCFA-containing supernatant. SCFAs, including acetate, propionate, and butyrate, were analyzed using gas chromatography-mass spectrometry (Hewlett Packard Model 7890) with a DB-FATWAX Ultra Inert column (Agilent, USA).

### Statistical Analysis

The results are expressed as mean ± standard deviation. One-way analysis of variance, followed by Tukey’s multiple comparisons test, was performed to compare multiple groups (GraphPad Prism software; GraphPad Prism, USA). Differences were considered significant at *p* < 0.05.

## Results

### Analysis of GJ Samples

The GJ samples (GJs) used in this study were produced and obtained from different regions (Table 1). Therefore, we first analyzed the content of biogenic amines (histamine and tyramine) and aflatoxins in the GJs. GJ1 contained 626.18 ± 0.64 mg/kg histamine and 437.41 ± 0.59 mg/kg tyramine; aflatoxin was not detected (Table 1). GJ2 contained 102.93 ± 0.99 mg/kg histamine, 429.95 ± 1.38 mg/kg tyramine, and 0.1 ± 0.02 μg/kg aflatoxin (Table 1). GJ3 contained 19.5 ± 1.74 mg/kg histamine and 89.15 ± 0.72 mg/kg tyramine; aflatoxin was not detected (Table 1). GJ4 contained 21.74 ± 1.80 mg/kg histamine and 83.62 ± 0.46 mg/kg tyramine; aflatoxin was not detected (Table 1). The salt content was 5.6472 ± 0.7058%, 7.8476 ± 0.3175%, 8.9668 ± 0.0544%, and 7.6483%± 0.4300% in GJ1, GJ2, GJ3, and GJ4, respectively (Table 1).

**Fig. 1:**
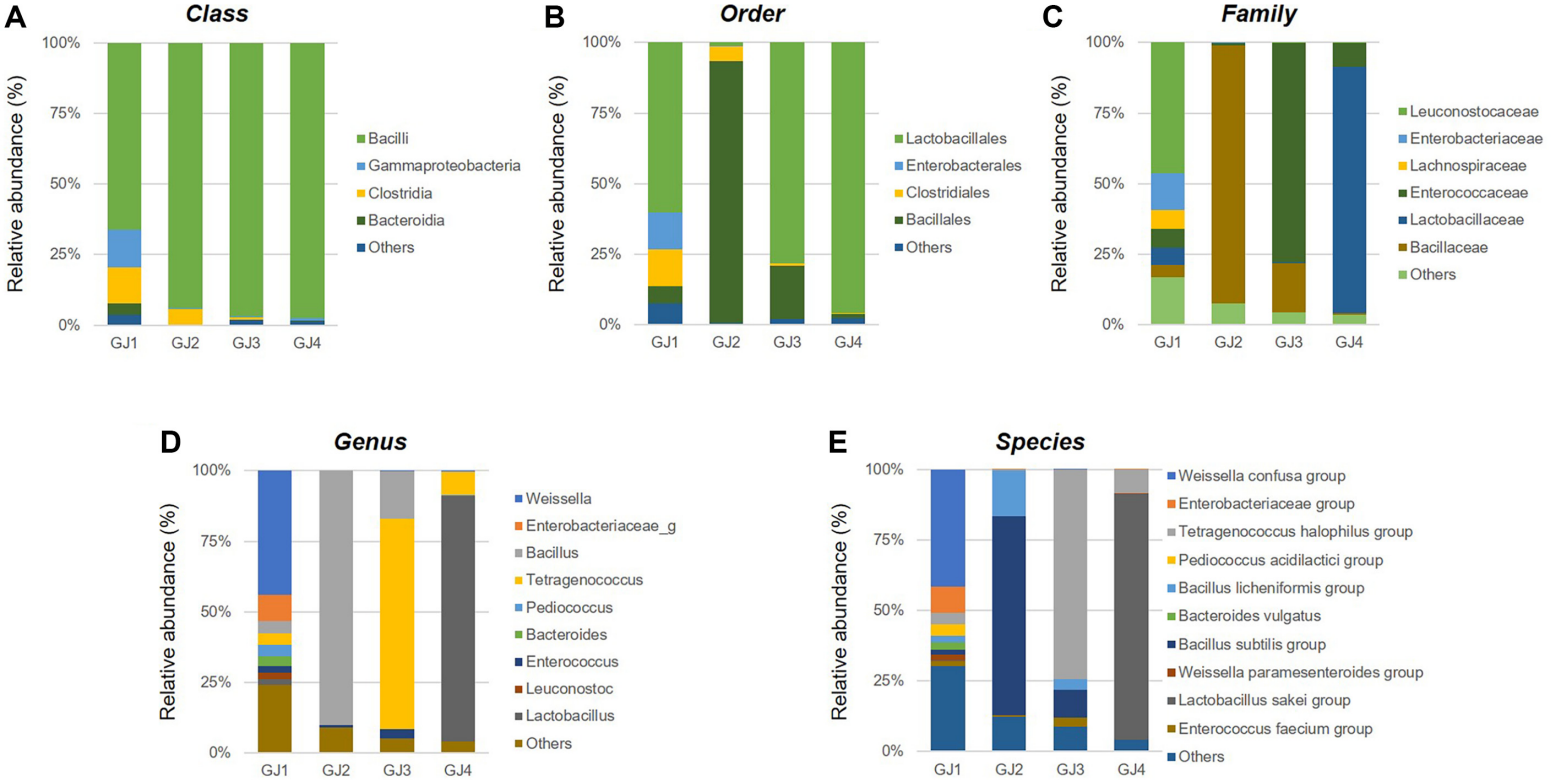
Microbial community analysis of GJs. Relative abundance (%) at the (**A**) class, (**B**) order, (**C**) family, (**D**) genus, (**E**) species levels.

We performed microbial analysis of the GJs using NGS to identify differences among the GJs. All GJs were dominated by Bacilli at the class level (Fig. 1A). Starting at the order level, the GJs showed differences in bacterial communities. At the order level, GJ1, GJ3, and GJ4 were dominated by Lactobacillales, and GJ2 was dominated by Bacillales (Fig. 1B). GJ1 was dominated by Leuconostocaceae, *Weissella*, and *Weissella* confusa group; GJ2 was dominated by Bacillaceae, *Bacillus*, and *Bacillus subtilis* group; GJ3 was dominated by *Enterococcaceae*, *Tetragenococcus*, and *Tetragenococcus halophilus* group; and GJ4 was dominated by *Lactobacillaceae*, *Lactobacillus*, and *Lactobacillus sakei* group at the family, genus, and species levels, respectively (Fig. 1C-1E). These results suggest that the BA, aflatoxin, and salt contents as well as bacterial communities of GJs may differ depending on the manufacturing region.

### GJ Reduces the Symptoms of DSS-Induced Colitis in Mice

**Fig. 2:**
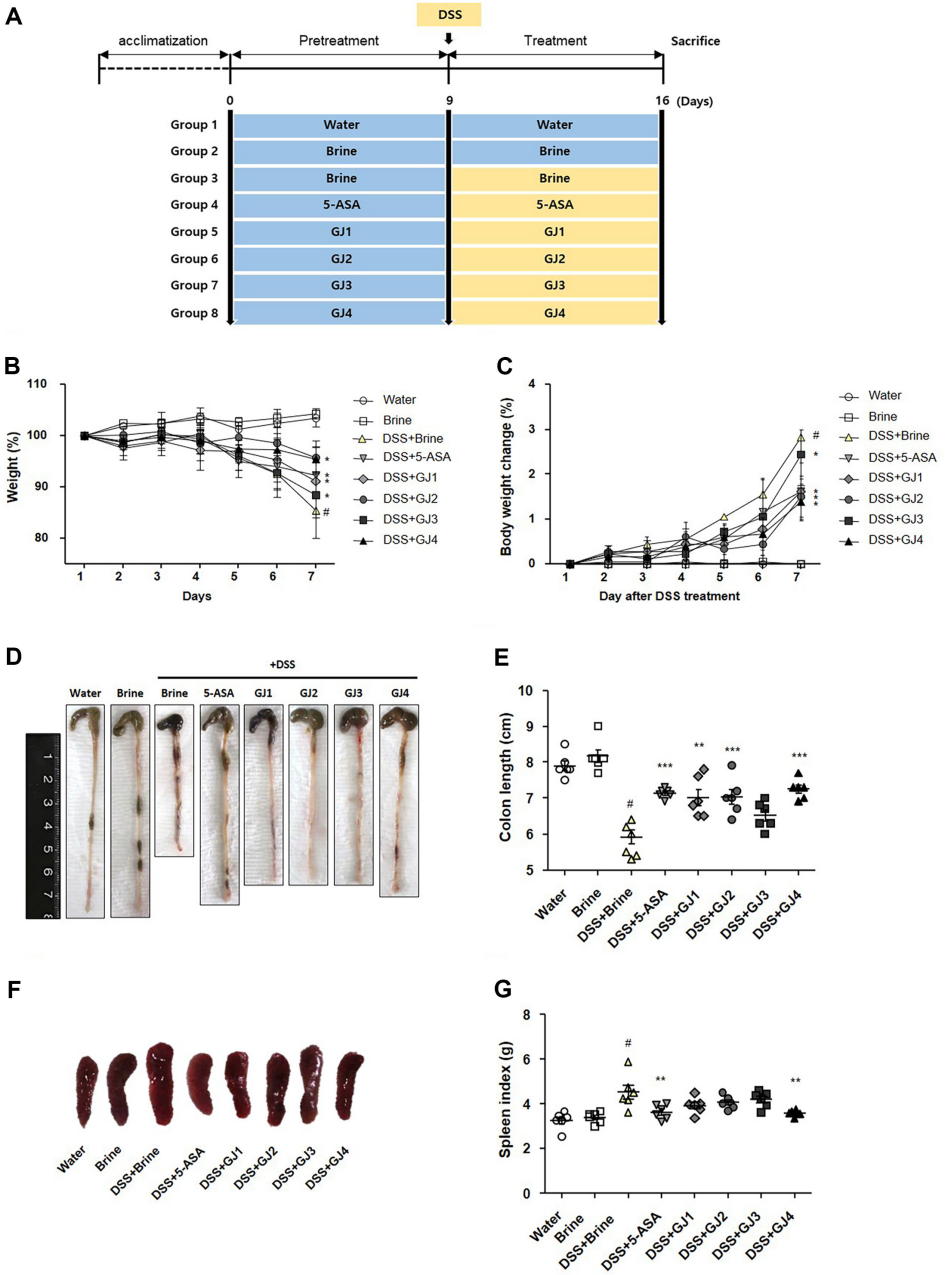
GJ reduces the symptoms of DSS-induced colitis mice. (**A**) Animal study design. (**B**) Body weight. (**C**) Body weight changes. (**D**) Representative picture of a colon. (**E**) Colon length. (**F**) Representative picture of a spleen. (**G**) Spleen index. All data are presented as mean ± SD (*n* = 6); #, *p* < 0.05 versus brine group; *, *p* < 0.05 versus DSS + brine group.

We evaluated the anti-inflammatory effects of GJ on intestinal inflammation using a DSS-induced colitis model (Fig. 2A). The acclimatized model mice were pre-treated with 5-ASA as the positive control or GJs (GJ1–GJ4) for 9 days and then treated with 5% DSS and 2% brine. The mice were then administered 5-ASA as the positive control or GJs (GJ1–GJ4) for 7 days. No differences were noted between the control groups administered water (water group) and 2% brine (brine group). After treatment with 5% DSS, the body weight of mice reduced compared with that of mice in the control groups (water and brine groups). However, the administration of 5-ASA and GJs restored these changes considerably (Fig. 2B and 2C). To confirm the anti-inflammatory effects of GJ on intestinal inflammation, we evaluated the symptoms of DSS-induced colitis based on the colon length and spleen weight. After treatment with 5% DSS, the colon length shortened and the spleen weight increased compared with those of mice in the control group (brine group). However, these changes were significantly reversed by the administration of 5-ASA and GJs (Fig. 2D-2G).

### GJ Restores the Histological Changes in Mice with DSS-Induced Colitis

**Fig. 3:**
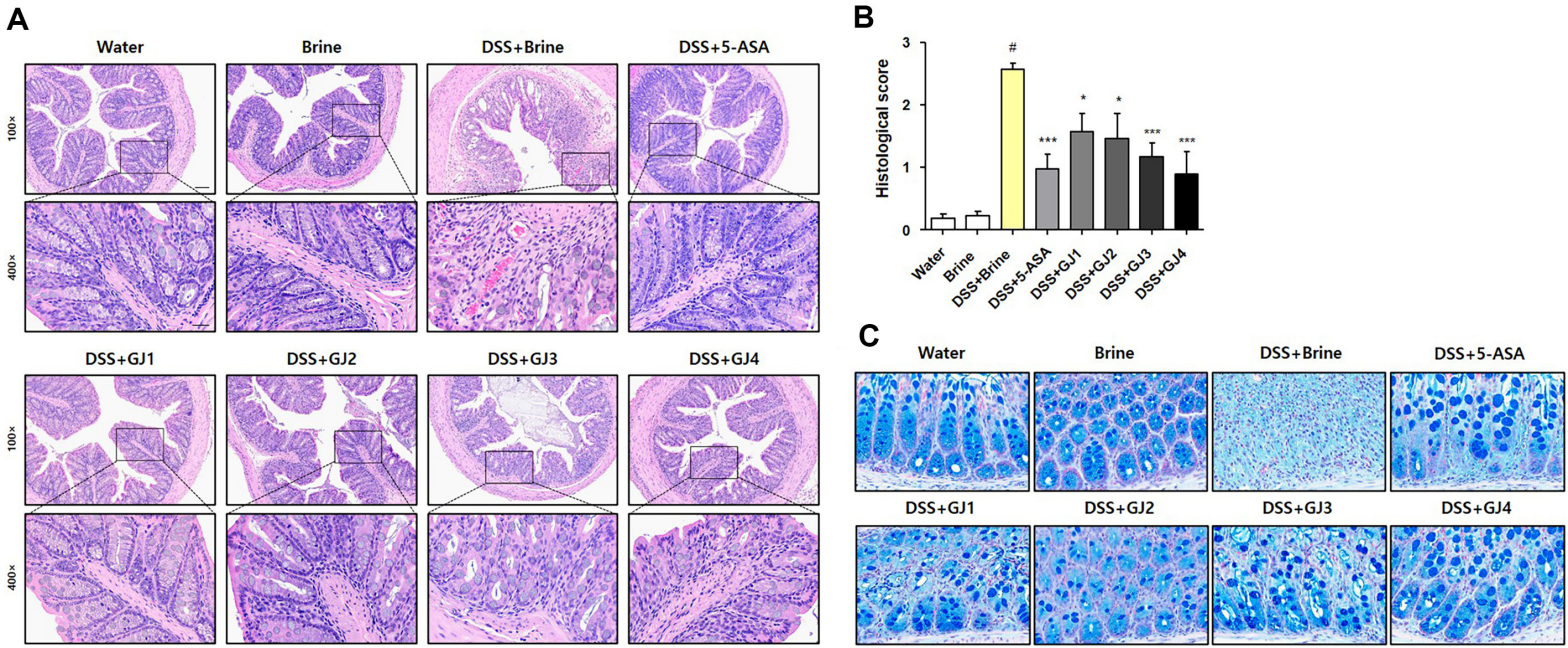
GJ administration reverts histological changes in mice with DSS-induced colitis. (**A**) Representative picture of H&E staining of the colon tissue. (**B**) Histological score. (**C**) Representative picture of Alcian blue staining of the colon tissue. All data are presented as mean ± SD (*n* = 6); #, *p* < 0.05 versus brine group; *, *p* < 0.05; ***, *p* < 0.001 versus DSS + brine group.

To evaluate the histological changes in mice with DSS-induced colitis, H&E staining was performed. After treatment with 5% DSS, goblet cells collapsed and inflammatory cell infiltration increased compared with those in the control groups (water and brine groups). However, these histological changes were considerably ameliorated by the administration of 5-ASA and GJs (Fig. 3A and 3B), as confirmed using Alcian blue staining. In mice treated with 5% DSS and 2% brine (DSS + brine group), goblet cells were destroyed compared to those in mice in the control groups (water and brine groups). However, the depletion of goblet cells was considerably ameliorated by the administration of 5-ASA and GJs (Fig. 3C).

### GJ Regulates Inflammatory Cytokine Concentrations at the mRNA and Protein Levels

**Fig. 4:**
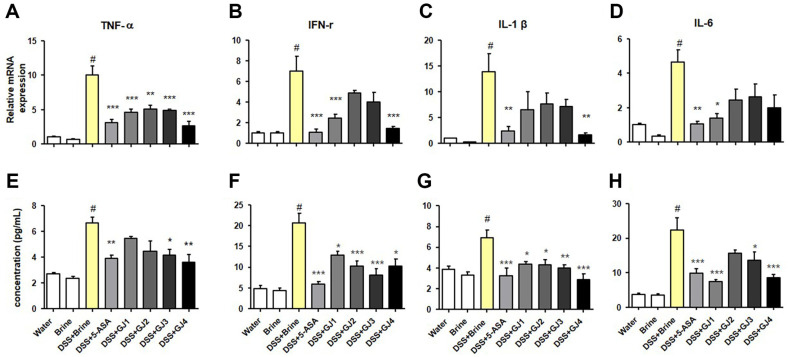
GJ regulates the levels of inflammatory cytokines. mRNA levels of (**A**) TNF-α, (**B**) IFN-γ, (**C**) IL-1β, and (**D**) IL-6 in the colon tissues. Serum protein levels of (**E**) TNF-α, (**F**) IFN-γ, (**G**) IL-1β, and (**H**) IL-6. All data are presented as mean ± SD (*n* = 6); #, *p* < 0.05 versus brine group; *, *p* < 0.05; **, *p* < 0.005; ***, *p* < 0.001 versus DSS + brine group.

Real-time PCR and ELISA were performed to determine the expression of inflammatory cytokines in mice with DSS-induced colitis. After treatment with 5% DSS, the mRNA expression of the inflammatory cytokines TNF-α, IFN-γ, IL-1β, and IL-6 increased compared with that in the control groups (water and brine groups). However, these changes were considerably ameliorated by the administration of 5-ASA and GJs (Fig. 4A-4D). These observations were also confirmed at the protein level using western blotting (Fig. 4E-4H).

### GJ Inhibits COX-2 and iNOS Expression through the MAPK Signaling Pathway and NF-κB Activation

**Fig. 5:**
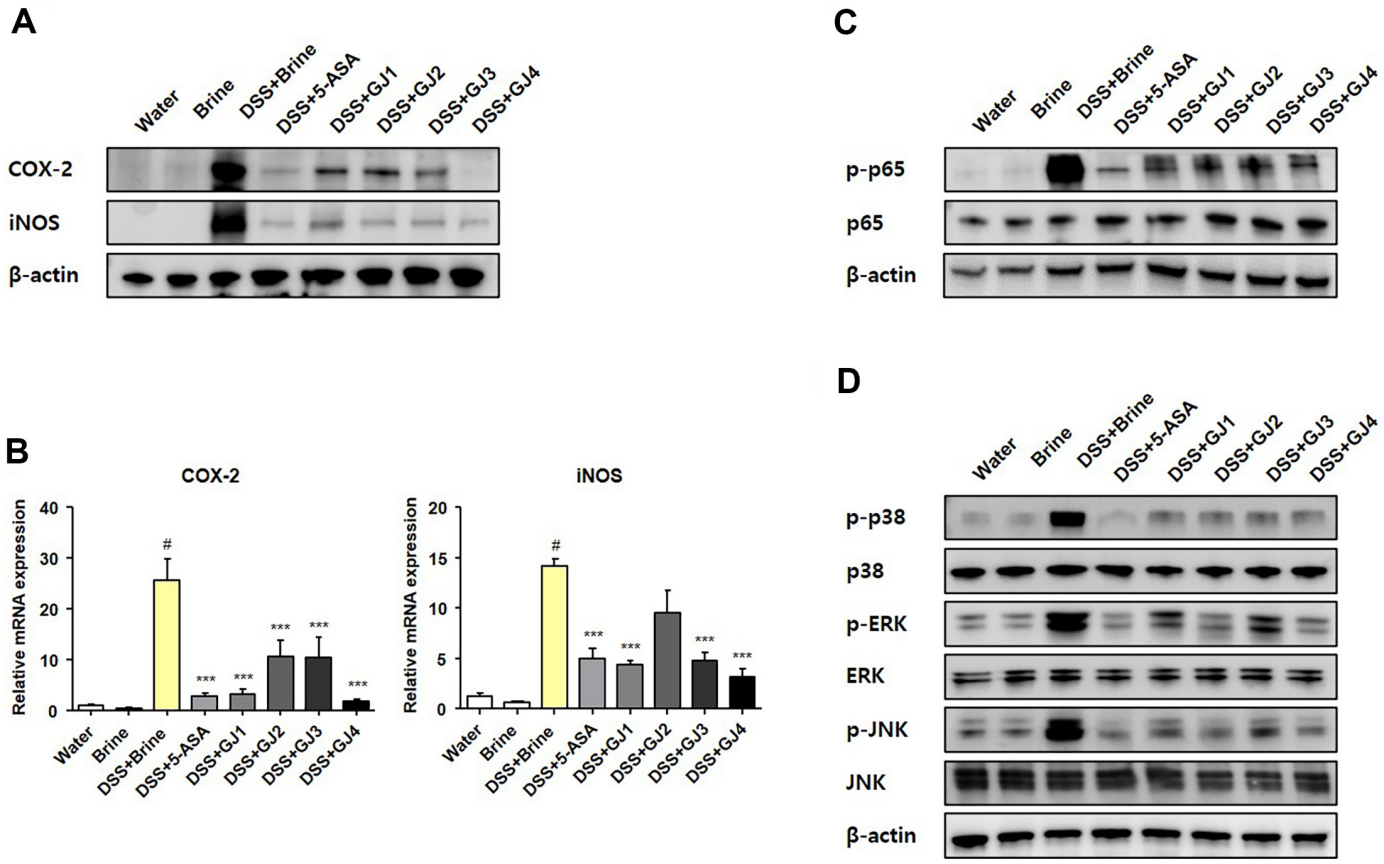
GJ regulates the MAPK and NF-κB signaling pathways in mice with DSS-induced colitis. (**A**) Protein and (**B**) mRNA levels of COX-2 and iNOS. Protein levels of (**C**) p-p65, p65, (**D**) p-p38, p38, p-ERK, ERK, p-JNK, and JNK. All data are presented as mean ± SD (*n* = 6); #, *p* < 0.05 versus brine group; ***, *p* < 0.001 versus DSS + brine group.

The overexpression of inflammatory mediators such as COX-2 and iNOS has been implicated in the development of DSS-induced colitis [17]. Inflammatory mediators are activated by the MAPK signaling pathway or NF-κB activation [18]. Therefore, we evaluated whether GJs regulate the expression of COX-2 and iNOS through the MAPK signaling pathway and NF-κB activation. The protein levels of COX-2 and iNOS were increased following 5% DSS treatment, and this effect was considerably reduced by the administration of 5-ASA and GJs (Fig. 5A). These results were confirmed at the mRNA level (Fig. 5B). We then investigated the expression of signaling molecules such as NF-κB and MAPK. The expression of phosphorylated p-65 (involved in the NF-κB signaling pathway) and phosphorylated p-38, ERK, and JNK (involved in MAPK signaling) proteins increased in response to treatment with 5% DSS. The increase in NF-κB and ERK levels was considerably ameliorated by the administration of 5-ASA and GJs (Fig. 5C and 5D).

### GJ Modulates the Tight Junction Protein Levels to Improve Intestinal Integrity

**Fig. 6:**
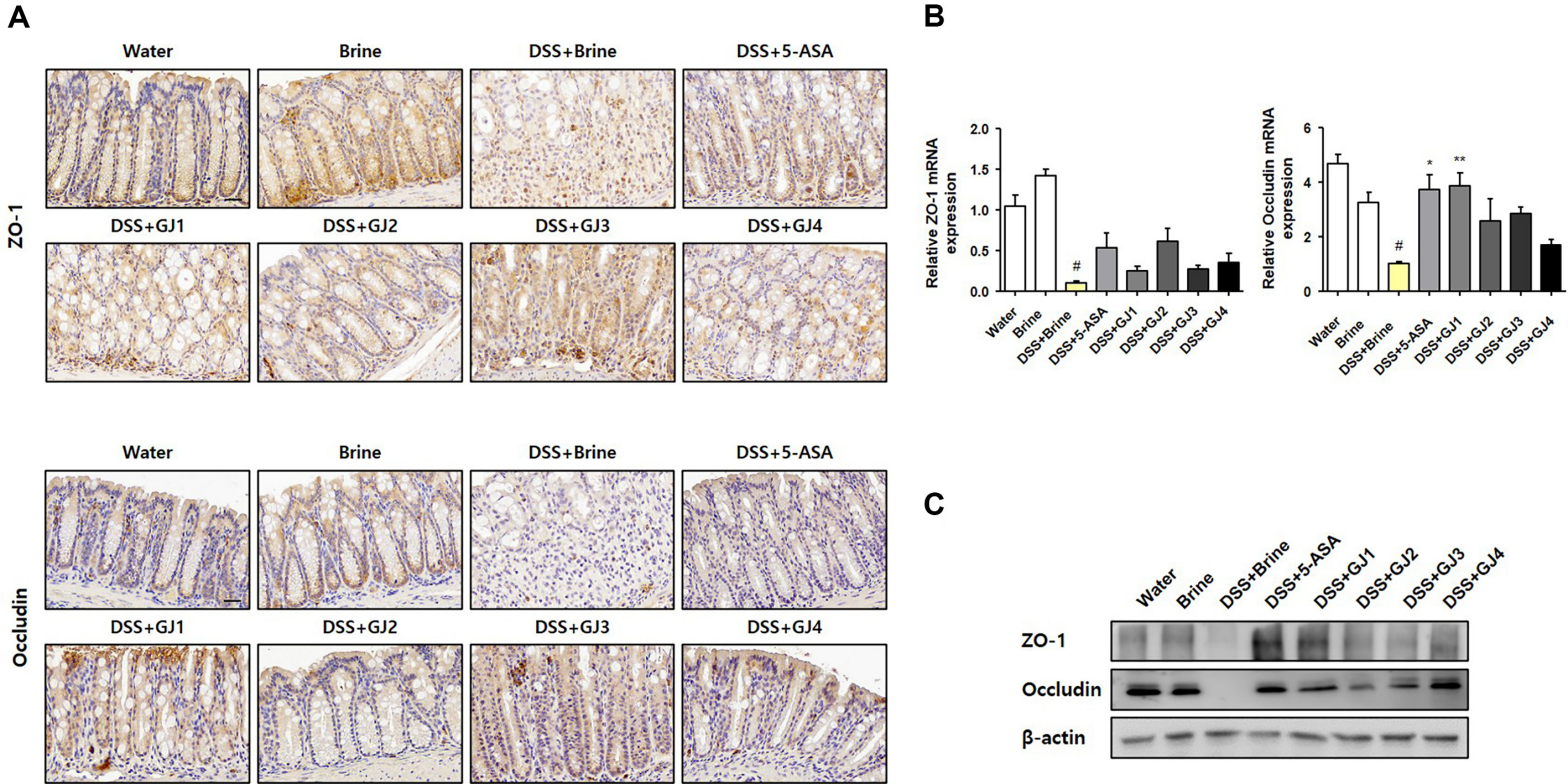
GJ modulates tight junction protein levels in mice with DSS-induced colitis. (**A**) Representative picture showing immunohistochemistry staining of the colon tissue. (**B**) mRNA levels and (**C**) protein levels of ZO-1 and occludin in the colon tissue. All data are presented as mean ± SD (*n* = 6); #, *p* < 0.05 versus brine group; *, *p* < 0.05; **, *p* < 0.005 versus DSS + brine group.

The normal intestinal epithelial barrier plays a crucial role in immune homeostasis and is regulated by tight junction proteins such as ZO-1 and occludin [19]. Therefore, we evaluated the expression of ZO-1 and occludin after DSS treatment using IHC staining. After treatment with 5% DSS, the protein levels of ZO-1 and occludin decreased compared with those in the control groups (water and brine groups). However, the decreased protein levels of ZO-1 and occludin were restored by the administration of 5-ASA and GJs (Fig. 6A). These effects were also confirmed at the protein and mRNA levels in the colon tissue (Fig. 6B and 6C).

### GJ Modulates the Fecal SCFA Concentrations

**Fig. 7:**
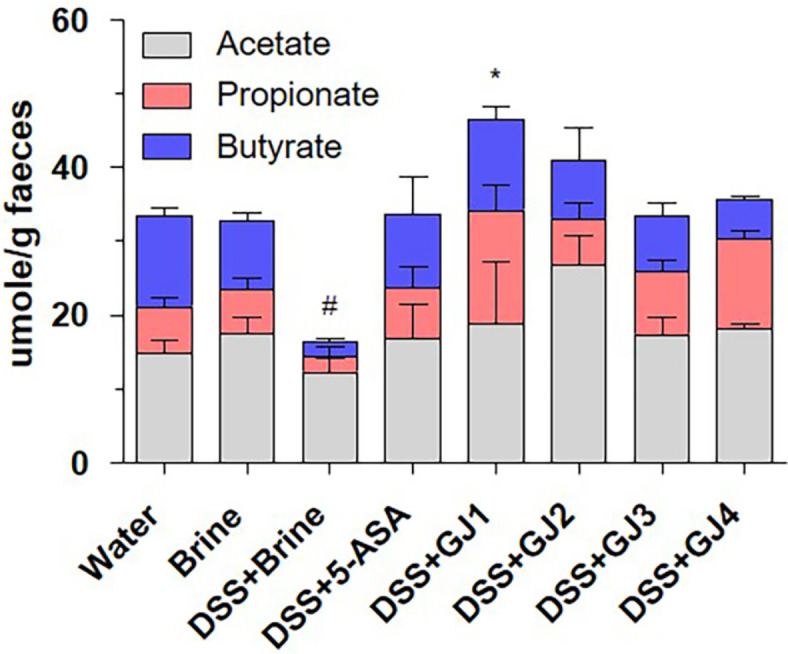
GJ modulates the intestinal microbiota and fecal SCFA levels. Levels of SCFAs (acetate, propionate, and butyrate) in fecal samples. All data are presented as mean ± SD (*n* = 6); #, *p* < 0.05 versus brine group; *, *p* < 0.05 versus DSS + brine group.

SCFAs are produced via anaerobic fermentation by the intestinal microbiota and are closely associated with intestinal health and pathology, including inflammation [20, 21]. Therefore, we evaluated the gut microbiota composition and SCFA (acetate, propionate, and butyrate) levels in the collected fecal samples. The butyrate level was markedly reduced in mice treated with 5% DSS compared with that in mice in the control groups (water and brine groups). However, the reduced butyrate level was considerably restored by the administration of 5-ASA and GJs (Fig. 7).

## Discussion

GJ, a traditional Korean fermented soy sauce, is commonly used as a liquid condiment in cooking worldwide [11]. It is generally produced by fermentation using one of the following two methods: traditional and commercial; its taste and quality differ depending on the microorganisms used [12]. Fermented foods are known to produce BA, which can cause neurotoxicity and raise safety concerns, and several studies have suggested acceptable levels of BA in foods, including histamine (100 mg/kg), tyramine (100–800 mg/kg), and β-phenylethylamine (30 mg/kg) [22, 23]. However, fermented foods contain abundant beneficial bacteria such as probiotics, which regulate intestinal microorganism composition and improve blood sugar control and immune function [24]. In addition, fermented foods have shown positive health benefits in aging, diabetes, and intestinal health [24]. In a previous study, two types of GJ (fermented soy sauce and sesame sauce) showed anti-inflammatory effects in mice with DSS-induced colitis [25]; however, the functional mechanism of action of GJ was not elucidated. Furthermore, comparative studies on microbial community and anti-inflammatory effects of GJs produced using traditional and commercial methods have not been conducted. Therefore, in the present study, we compared the differences in BA contents and microbial communities between traditionally and commercially manufactured GJs and confirmed their anti-inflammatory effect in mice with DSS-induced colitis.

We first analyzed the components of traditionally and commercially manufactured GJs. The content of histamine and tyramine in GJ2–GJ4 was in the range of 19.5 ± 1.74 to 102.93 ± 0.99 mg/kg and 89.15 ± 0.72 to 429.95 ± 1.38 mg/kg, respectively (Table 1), which were confirmed to be acceptable levels in foods. However, the content of histamine and tyramine in GJ1 was 626.18 ± 0.64 and 437.41 ± 0.59 mg/kg, respectively; the histamine content was slightly higher than the acceptable levels in food [22]. However, considering the low intake per serving, the risk of BA intake in GJ1 may not be significant [23]. It is known that high-salt fermented foods are abundant in beneficial bacteria such as *Bacillus* spp. and lactic acid bacteria [26]. Therefore, we analyzed the microbial community of GJs. As expected, all GJs, regardless of the manufacturing methods and regions, were dominated by bacilli, which are beneficial bacteria at the order level. However, depending on the manufacturing regions, GJs showed differences in microbial communities at the order, family, genus, and species levels (Fig. 1). These results indicate that GJ contains abundant beneficial bacteria, including probiotic strains. Furthermore, they highlight the variability in BA content and microbial community structure within GJs, suggesting a potential influence of geographical factors on the manufacturing process.

We used a DSS-induced colitis model to assess the anti-inflammatory effects of GJs due to differences in BA content and microbial community structure. In animal experiments, the administration of 5% DSS significantly aggravated the symptoms of DSS-induced colitis, such as body weight loss, colon length shortening, and spleen weight increase. However, administration of GJs for 16 days suppressed the symptoms of DSS-induced colitis, similar to that in the positive control group (5-ASA) (Fig. 2). Administration of 5% DSS also caused histological changes, such as goblet-cell depletion and increased inflammatory cell infiltration. These histological changes were considerably ameliorated by the administration of GJs, similar to that in the positive control group (5-ASA)(Fig. 3). Notably, the effects of traditionally prepared GJs (GJ1–3) in alleviating DSS-induced colitis symptoms were similar to or better than the effects of the commercial GJ (G4).

Inflammatory cytokines, such as TNF-α, IFN-γ, IL-1β, and IL-6, play a crucial role in inflammatory responses [27]. The levels of inflammatory cytokines are known to increase in animal models of DSS-induced colitis and are closely associated with IBD development [28]. Fermented foods are recognized for their abundance of beneficial bacteria, including probiotic strains [26]. Probiotics primarily exert immune-modulating and anti-inflammatory effects, achieved through the regulation of pro-inflammatory cytokine signaling pathways, such as the TNF-alpha signaling pathway [29,30]. Therefore, we investigated whether GJ, which contains abundant probiotic strains, affects the increased level of inflammatory cytokines observed in animal models of DSS-induced colitis. Consistent with the findings of previous studies, in this study, the serum concentration and mRNA levels of inflammatory cytokines significantly increased following 5% DSS administration in the DSS treatment groups (DSS + Brine). However, the administration of GJs (GJ1–GJ4) decreased the serum concentration and mRNA levels of inflammatory cytokines, similar to that in the positive control group (5-ASA) (Fig. 4). In the inflammatory response, the upregulation of expression of the inflammatory mediators iNOS and COX-2 is regulated by NF-κB activation and the MAPK signaling pathway [17, 18]. Probiotics are known to inhibit NF-kB activation and reduce p-38 MAPK phosphorylation and iNOS expression [31]. Therefore, we investigated whether GJ administration affects inflammatory mediator expression, NF-κB activation, and the MAPK signaling pathway in the DSS-induced colitis animal model. The administration of 5% DSS significantly increased the protein and mRNA levels of iNOS and COX-2 in mice. Furthermore, the administration of 5% DSS significantly stimulated the activation of NF-κB and the MAPK signaling pathway and increased the expression of phosphorylated p65, p38, ERK, and JNK (Fig. 5). However, these effects were considerably attenuated by GJ administration (GJ1–GJ4). These results suggest that GJs (GJ1–GJ4) alleviate the symptoms of DSS-induced colitis by inhibiting the expression of inflammatory cytokines and mediators via NF-κB activation and the MAPK signaling pathway.

Epithelial tight junction proteins, including occludin and ZO-1, play key roles in maintaining the integrity of the intestinal barrier [19, 32]. However, downregulation of epithelial tight junction protein expression leads to the disruption and loss of function of the epithelial barrier, ultimately causing IBD. In the present study, we observed that the administration of 5% DSS significantly reduced the expression of ZO-1 and occludin. However, the administration of GJs (GJ1–GJ4) considerably restored their levels (Fig. 6). These observations were confirmed at the protein and mRNA levels using animal tissues.

IBD development is closely related to an imbalance in the gut microbiota composition [33], which regulates metabolic, endocrine, and immune functions [34]. The gut microbiota, including more than 1,000 bacterial species, produces SCFAs via dietary fiber fermentation [9]. SCFAs play crucial roles in human gut health by regulating immune functions [9, 33, 34]. Furthermore, the increase in inflammatory cell numbers in the gastrointestinal tract of patients with IBD is closely associated with a decrease in SCFA levels [35]. Recent studies have shown that the gut microbiota composition is influenced by diet, and fermented foods containing probiotics improve human gut health by affecting the gut microbiota composition and SCFA production [36, 37]. Furthermore, the ratio of SCFAs depends on not only the gut microbiota composition but also the fermentation of dietary fiber [9]. In this study, we observed that the administration of 5% DSS reduced the total SCFA levels, including acetic acid, propionic acid, and butyric acid levels, and this effect was significantly restored by the administration of GJs (GJ1–GJ4) (Fig. 7).

In conclusion, we observed that regardless of the manufacturing method, GJ administration significantly alleviated the symptoms of DSS-induced colitis, such as body weight loss, colonic shortening, enlarged spleen, and histological changes, in a mouse model. We also observed that GJ regulates the expression of inflammatory cytokines and inhibits the protein expression of COX-2 and iNOS through the activation of NF-κB and MAPK signaling pathways. Furthermore, GJ modulated tight junction protein and fecal SCFA concentrations. On the basis of these findings, GJ can ameliorate DSS-induced symptoms by regulating NF-κB and MAPK signaling and modulating SCFA levels. Therefore, we propose that GJ holds promise as a fermented food option for preventing inflammatory diseases such as colitis.

## Figures and Tables

**Table 1:** BA, aflatoxin, and salt content of GJs.

Ganjang	Region	Biogenic amine		Sodium (wt%)	Total Aflatoxin
		Histamine	Tyramine		
GJ1	Sunchang-gun, Jeollabuk-do	626.18 ± 0.64	437.41 ± 0.59	5.6472 ± 0.7058	N.D
GJ2	Paju-si, Gyeonggi-do	102.93 ± 0.99	429.95 ± 1.38	7.8476 ± 0.3175	0.1 ± 0.02
GJ3	Cheongwon-gu, Chungcheonbuk-do	19.5 ± 1.74	89.15 ± 0.72	8.9688 ± 0.0544	N.D
GJ4	Sunchang-gun, Jeollabuk-do (Commercial company)	21.74 ± 1.80	83.62 ± 0.46	7.6483 ± 0.4300	N.D
